# Common Medical Comorbidities, Demographic Factors and Levels of Plasma Biomarkers of Alzheimer’s Disease and Neurodegeneration in Black/African American Older Adults

**DOI:** 10.3390/biom16050747

**Published:** 2026-05-19

**Authors:** Rosie E. Curiel Cid, Alexandra Ortega, Kirsten Crenshaw, Juan Pablo de Rivero Vaccari, Minerva M. Carrasquillo, David Vaillancourt, D. Diane Zheng, Dylan Hinton, Efrosyni Sfakianaki, Elizabeth A. Crocco, Heidi Casellas, Lindsey A. Kuchenbecker, Sofia Ramirez, Tan Abascal, Triana Abel, Wei-En Wang, Ranjan Duara, Nilüfer Ertekin-Taner, David A. Loewenstein

**Affiliations:** 1 Center for Cognitive Neuroscience and Aging (CNSA), Miller School of Medicine, University of Miami, Miami, FL 33146, USA; axo623@med.miami.edu (A.O.); kah196@med.miami.edu (K.C.); dzheng@med.miami.edu (D.D.Z.); dmh275@med.miami.edu (D.H.); ecrocco@med.miami.edu (E.A.C.); sxr3624@med.miami.edu (S.R.); taa115@med.miami.edu (T.A.); tma1313@med.miami.edu (T.A.); dloewenstein@med.miami.edu (D.A.L.); 2 Department of Neurological Surgery and The Miami Project to Cure Paralysis, Miller School of Medicine, University of Miami, Miami, FL 33146, USA; jderivero@med.miami.edu; 3 Department of Neuroscience, Mayo Clinic, Jacksonville, FL 55905, USA; carrasquillo.minerva@mayo.edu (M.M.C.); casellas.heidi@mayo.edu (H.C.); kuchenbecker.lindsey@mayo.edu (L.A.K.); taner.nilufer@mayo.edu (N.E.-T.); 4 Department of Applied Physiology and Kinesiology, University of Florida, Gainesville, FL 32611, USA; vcourt@ufl.edu (D.V.); weienwang@ufl.edu (W.-E.W.); 5 Division of Nuclear Medicine, Department of Radiology, Miller School of Medicine, University of Miami, Miami, FL 33146, USA; esfakianaki@med.miami.edu; 6 Wien Center for Alzheimer’s Disease and Memory Disorders, Mount Sinai Medical Center, Miami Beach, FL 33140, USA; ranjan.duara@msmc.com

**Keywords:** plasma, comorbidities, biomarkers, MCI, Alzheimer’s disease, African American

## Abstract

Emerging evidence suggests that systemic physiological factors may influence plasma biomarker concentrations of Alzheimer’s disease (AD) and related neurodegenerative processes, potentially affecting their specificity for central nervous system pathology. This study examined the relationship of demographic factors and medical comorbidities with plasma biomarkers of AD and neurodegeneration in a community-dwelling cohort of Black/African American (B/AA) older adults (N = 141). Participants underwent plasma assessment of phosphorylated tau at threonine 217 (p-Tau217), glial fibrillary acidic protein (GFAP), and neurofilament light chain (NfL). Results showed associations between plasma p-Tau217 and amyloid PET positivity, and significant intercorrelations among p-Tau217, GFAP, and NfL. Stepwise regression models incorporated demographics, amyloid PET status, and laboratory measures of renal, metabolic, and lipid function as predictors for each biomarker. p-Tau217 was primarily predicted by amyloid PET and renal function; GFAP by age and sex; and NfL by renal function, age, and sex. Findings indicate plasma biomarker concentrations in B/AA older adults reflect both central AD-related pathology and systemic physiological factors, particularly renal function, and demographic influences. Results underscore the importance of accounting for comorbid medical conditions and demographic characteristics when interpreting blood-based biomarkers and highlight the need for comprehensive medical phenotyping to improve diagnostic specificity and clinical utility.

## 1. Introduction

Alzheimer’s disease (AD) and AD-related dementia (ADRD) represent a significant and growing global public health challenge, with their prevalence rising alongside a rapidly aging and increasingly diverse worldwide population. In the United States, the prevalence of clinical dementia is notably higher in non-Hispanic Black adults compared to non-Hispanic White adults, with estimates suggesting that older Black/African American (B/AA) individuals bear a disproportionate burden of cognitive impairment and dementia [1,2,3,4]. Further, the population of older B/AA adults is expected to represent approximately 12% of the nation’s population by 2060, which is a 192% increase compared to 2020 [5]. This underscores the critical need for scientific efforts focused on improving diagnostic strategies to thoughtfully examine the factors contributing to higher dementia risk among B/AA older adults. The disproportionate burden of AD and ADRD in B/AA individuals has been thought to result from a complex interplay of structural and social determinants of health, environmental exposure, and biological factors [5,6]. For instance, B/AA older adults experience a higher prevalence of multiple medical comorbidities relative to other racial and ethnic groups [7,8]. This poses a significant challenge, as common medical conditions including cerebrovascular disease, chronic kidney disease, and metabolic disorders are known to interact with and augment the risk for AD/ADRD [9]. The high prevalence of systemic medical conditions in B/AA older adults may not only increase dementia risk but may also complicate the interpretation of emerging blood-based biomarkers (BBMs) of AD/ADRD.

The development of BBMs that can detect the buildup of hallmark AD/ADRD pathological processes such as abnormal amyloid-β (Aβ) proteins, AD-specific phosphorylated tau, damage to neurons, and astrocyte and microglial activation offers the promise of a cost-effective, less invasive, and highly scalable method for improved diagnostic accuracy, and monitoring of AD/ADRD. Among the emerging plasma biomarkers, phosphorylated tau (pTau), particularly p-Tau217, along with neurofilament light chain (NfL), and glial fibrillary acidic protein (GFAP), are garnering considerable attention for their potential roles in helping to detect the presence of neurodegenerative disease and its progression. Plasma p-Tau217 is one of the most promising BBMs that are closely associated with an AD diagnosis due to its correlation with AD-specific tau pathology, and strong concordance with key pathological proteins (amyloid and tau) assessed by cerebrospinal fluid (CSF) and positron emission tomography (PET) [10,11]. However, research related to these novel BBMs in B/AA older adults remains relatively limited, and there are several factors that warrant consideration when using BBMs to diagnose AD/ADRD.

An important factor to consider is the individual’s overall systemic health and its potential influence on BBMs of AD/ADRD. Emerging evidence indicates that comorbid medical conditions can affect BBM levels [11,12,13,14,15,16,17,18,19,20,21]. Thus, understanding the influence of prevalent medical comorbidities on BBMs of AD/ADRD is essential to avoid diagnostic misclassification and improve differential diagnosis. For example, impaired renal function has been associated with significantly elevated levels across a range of biomarkers, including Aβ40, Aβ42, NfL, GFAP, and p-Tau217 [13,14]. Specifically, a lower estimated glomerular filtration rate (eGFR) and higher creatinine, which is generally attributed to reduced renal clearance, have been associated with elevated levels of plasma p-Tau217 and neurodegeneration markers such as NfL [14,22]. Importantly, impaired renal function remained associated with elevated biomarker levels, such p-Tau217, even after adjusting for demographics, APOE ε4 status, and progression to dementia, suggesting that elevated biomarker concentrations in the context of impaired renal function may reflect reduced peripheral clearance rather than increased central AD pathology [23,24,25,26]. However, recent work by Arslan and colleagues [27] reported that although renal function is associated with plasma biomarker concentrations, adjustment for eGFR provided negligible improvement in predicting amyloid-beta positivity, particularly within the range of normal-to-mild renal impairments.

The presence of abnormal metabolic profiles, including elevated fasting glucose and hemoglobin A1C (HbA1C), indicators of diabetes and measures of glucose control, has also been significantly related to multiple BBMs including Aβ40, Aβ42, total tau, and NfL in a large and ethnically diverse sample [13]. Few studies have systematically examined how prevalent medical comorbidities may influence BBM concentrations among B/AA older adults specifically; however, in one study examining p-Tau217 in a B/AA population, a history of diabetes was not significantly associated with plasma p-Tau217, after adjustments for age and sex [23]. A diagnosis of dyslipidemia that usually includes abnormal triglycerides, high-density lipoprotein (HDL), and total cholesterol showed significant correlations with Aβ40, Aβ42 and total tau [13]. Importantly, evidence regarding the influence of metabolic and systemic factors on plasma biomarker concentrations remains limited and, in some cases, inconsistent, underscoring the need for further investigations.

As plasma-based biomarkers gain clinical momentum for AD/ADRD detection and monitoring, emerging evidence suggests that systemic medical conditions may confound plasma biomarker concentrations. Investigating how prevalent medical conditions shape these profiles is essential for ensuring diagnostic reliability. This is particularly critical for understudied populations such as community-dwelling B/AA older adults who experience both elevated dementia risk and a high burden of comorbid conditions. This approach is needed to generate normative references and inform interpretative frameworks that ensure these biomarkers are accurate, reliable, and clinically meaningful across diverse populations. Accordingly, this study addresses an important knowledge gap by evaluating the effects of three highly prevalent comorbidities—dyslipidemia, diabetes, and chronic kidney disease (CKD)—measured both by self-report as well as continuous laboratory values as objective proxies for these conditions, in a large community-based sample of B/AA older adults.

## 2. Materials and Methods

### 2.1. Participants

The study cohort comprised 141 community-dwelling older adults who self-identified as Black/African American (B/AA) and were enrolled through an Institutional Review Board-approved protocol at the University of Miami. Participants were recruited from community-based settings across South Florida. The mean age was 68.2 years (SD = 6.0; range = 59–88). The sample included 82 females (58.6%). All included participants had available amyloid PET imaging, plasma biomarker data, and clinical laboratory measures. Exclusion criteria included active substance use disorder and major neuropsychiatric conditions that could interfere with study participation or the validity of cognitive and biomarker assessments. Participants with missing core imaging or biomarker data were excluded from analytic models. Global cognitive functioning was largely preserved (MMSE: M = 27.3, SD = 2.4). Following a comprehensive clinical evaluation comprising an extensive neuropsychological battery and informant interviews using the Clinical Dementia Rating (CDR) scale, cognitive diagnoses were established via multidisciplinary consensus. The resulting sample (N = 141) consisted of individuals classified as cognitively normal (n = 44), those meeting criteria for Mild Cognitive Impairment (MCI; n = 59), and a subgroup exhibiting clinical impairment who did not meet formal MCI diagnostic criteria (n = 38). Approximately 35.7% of participants were carriers of the apolipoprotein E ε4 (APOE ε4) allele.

### 2.2. Measures

#### 2.2.1. Plasma Biomarkers of AD and Neurodegeneration

Blood samples were collected in EDTA tubes and centrifuged within one hour of venipuncture at room temperature to isolate plasma. Aliquots were stored at −80 °C until analysis. Quantification of plasma biomarkers was performed at the Mayo Clinic, Jacksonville.

Plasma phosphorylated tau at threonine 217 (p-Tau217; ALZPath) was measured using the Quanterix HD-X Analyzer (Quanterix Corporation, Billerica, MA, USA) with the p-Tau217 SiMoA assay following manufacturer protocols. Samples were assayed in duplicate and averaged, with concentrations reported in pg/mL. Additional biomarkers included glial fibrillary acidic protein (GFAP) and neurofilament light chain (NfL). All biomarker values were required to meet a coefficient of variation < 20% to ensure analytic reliability, consistent with established assay quality control standards.

#### 2.2.2. Genotyping

*APOE* genotyping for 2/2, 2/3, 3/4 and 4/4 was conducted at Mayo Clinic, Jacksonville, using TaqMan SNP Genotyping Assays targeting rs7412 and rs429358 (Thermo Fisher Scientific, Waltham, MA, USA) on the QuantStudio 7 Flex Real-Time PCR system (Applied Biosystems, CA, USA). Genotypes were classified as ε4 carriers (one or two ε4 alleles) versus non-carriers.

#### 2.2.3. Medical Comorbidities and Clinical Blood Measures

Fasting clinical blood assays assessing renal function (eGFR, blood urea nitrogen [BUN], creatinine), metabolic status (fasting glucose, hemoglobin A1c [HbA1c]), and lipid profiles (total cholesterol, triglycerides, and LDL) were performed locally by the University of Miami Diabetes Research Center. In the absence of accessible formal medical records for this community-based cohort, continuous laboratory measures were used as objective proxies for these conditions. These measures are well-established and provide greater diagnostic precision than self-report data, which are more commonly relied upon in community-based research.

### 2.3. Amyloid-β PET Imaging and Visual Rating of Images

All participants underwent Amyloid-β PET imaging which was performed with either Florbetaben or Florbetapir. Images were visually rated by an experienced reader blinded to cognitive and clinical data, using a methodology similar to that described by Seibyl et al. Images were displayed using a reader-adjustable gray scale to provide optimal discrimination of the cerebellar gray matter from white matter. Subsequently, all the Aβ-PET scan slices were viewed using this gray scale adjustment. Tracer uptake was assessed in six cortical regions (orbitofrontal, frontal, parietal, lateral temporal, occipital and precuneus/posterior cingulate cortex), combining values from the left and right hemispheres. A final dichotomous (A+ versus A−) determination was rendered. Prior work from Loewenstein and colleagues reports reliabilities for amyloid visual reads between independent raters [28].

### 2.4. Statistical Analyses

Descriptive statistics characterized demographic, biomarker, and clinical variables. A covariance matrix was employed to determine the relationship between positive and negative amyloid PET readings and plasma log10-transformed p-Tau217, GFAP, and NfL. The associations between these biomarkers were also examined. Stepwise linear regression models with listwise deletion were used to identify further independent predictors of plasma p-Tau217, GFAP, and NfL concentrations. Listwise deletion was used to address missing data, resulting in the exclusion of 10 cases. Missingness was due to unavailable laboratory values. There was no discernible pattern of missingness, and these cases did not differ from the 131 participants retained for analysis (out of 141 total). Biomarker variables and clinical blood measures (HbA1c, BUN, creatinine, eGFR, total cholesterol, LDL, triglycerides) were log10-transformed to address skewness and improve normality. Predictors included age (continuous), sex (male = 0, female = 1), *APOE* ε4 carrier status (non-carrier = 0, carrier = 1) and amyloid PET read (negative = 0 and positive = 1). Model fit was evaluated using R^2^, adjusted R^2^, and standard error of estimate (SEE). Incremental variance explained at each step was assessed using ΔR^2^. Statistical significance was set at *p* < 0.05.

Tables 1–5 summarize cohort characteristics, biomarker distributions, and regression model results.

## 3. Results

### 3.1. Participant Characteristics

Demographic and clinical characteristics of the cohort are summarized in Table 1 and Table 2. The mean age of the sample was 68.2 (SD = 6.0; range = 59–88) years and 58.6% were female. Participants were cognitively intact on average (MMSE: M = 27.3, SD = 2.4), with 35.7% carrying at least one APOE ε4 allele. In this community-based sample, 18.4% of participants were amyloid PET positive.

### 3.2. Biomarker Distributions

Plasma biomarker measures were log10-transformed to address skewness and improve normality, consistent with current practices in the field. Plasma biomarker distributions are presented in Table 1. Given biomarker concentrations were skewed, medians are presented with the interquartile at the 25th and 75th percentiles respectively. Median concentrations indicated measurable levels of AD-related and neurodegenerative markers, including p-Tau217 [median 0.27 (IQR 0.19–0.410 pg/mL)], GFAP [median 78.32 (IQR 55.94–110.78 pg/mL)], and NfL [median = 9.81 (IQR 7.04–14.18 pg/mL)]. Mean values and standard deviations are presented in Table 1.

### 3.3. Medical Comorbidities

Clinical indicators of prevalent medical comorbidities that may confound plasma AD biomarker interpretation were examined, with particular focus on renal function, metabolic status, and lipid levels. Clinical blood measures reflected substantial variability in renal function, metabolic status, and lipid profiles within the cohort. Renal indices included the estimated glomerular filtration rate (eGFR), serum creatinine, and blood urea nitrogen (BUN). The mean eGFR for the cohort was 78.7 mL/min/1.73 m^2^ (SD = 24.1), with a minimum value of 9.5. Mean serum creatinine was 1.08 mg/dL (SD = 0.59). Metabolic profiles were also evaluated; hemoglobin A1c (HbA1c) was 6.01 (SD = 1.02). Finally, lipid profiles included measures of total cholesterol, triglycerides, and low-density lipoprotein (LDL). The mean total cholesterol was 173.4 mg/dL (SD = 42.0), triglycerides was 104.8 mg/dL (SD = 60.8), and LDL was 93.71 mg/dL (SD = 35.3). These values were also log10-transformed to address skewness and improve normality for subsequent data analyses.

We examined associations among LDL, HbA1c, eGFR, BUN, and creatinine to explore the potential multicollinearity among renal and metabolic measures. As expected, strong correlations were observed among renal indices, including eGFR with creatinine (r = −0.928, *p* < 0.001) and BUN (r = −0.638, *p* < 0.001), as well as between BUN and creatinine (r = 0.615, *p* < 0.001). In contrast, eGFR and BUN were not correlated with HbA1c or lipid measures. Creatinine showed statistically significant but small negative correlations with total cholesterol (r = −0.232, *p* = 0.008) and LDL (r = −0.182, *p* = 0.035), which are negligible in effect size and not clinically meaningful. HbA1c was not associated with any other laboratory measures. As expected, total cholesterol and LDL were highly correlated (r = 0.851, *p* < 0.001), with more modest associations observed between cholesterol and triglycerides (r = 0.353, *p* < 0.001) and between LDL and triglycerides (r = 0.252, *p* = 0.004).

### 3.4. Intercorrelations Between Plasma Biomarkers and Amyloid PET Positivity

Amyloid PET positivity (coded 0 = amyloid negative, 1 = amyloid positive) was significantly correlated with plasma p-Tau217 (r = 0.53, *p* < 0.001) and GFAP (r = 0.36, *p* < 0.001), and modestly correlated with NfL (r = 0.23, *p* = 0.006). Plasma p-Tau217 also demonstrated significant correlations with GFAP (r = 0.42, *p* < 0.001) and NfL (r = 0.46, *p* < 0.001).

### 3.5. Predictors of Plasma Biomarkers of AD and Neurodegeneration

A major goal of this study was to determine the extent to which blood-based biomarkers of AD and neurodegeneration were associated with demographic characteristics, common medical comorbidities, and *APOE* e4 carrier status above and beyond the effects of amyloid PET status alone in our B/AA older adult population.

### 3.6. Predictors of Plasma p-Tau217

**Figure 1:**
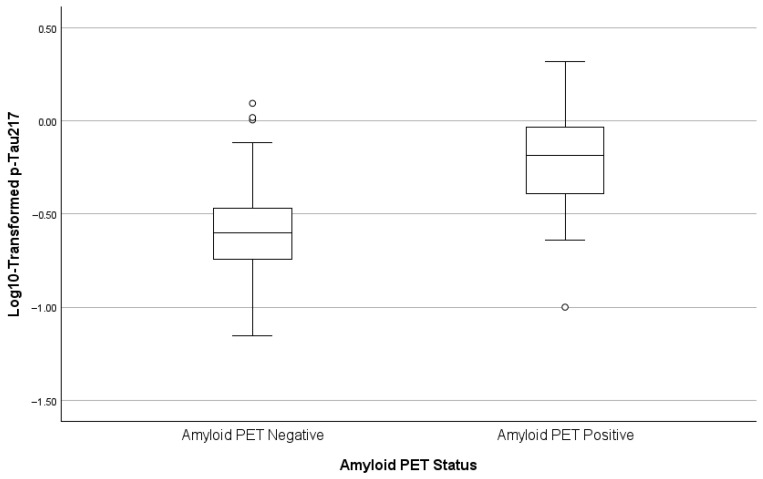
Box plots of p-Tau217 by amyloid PET status.

**Figure 2:**
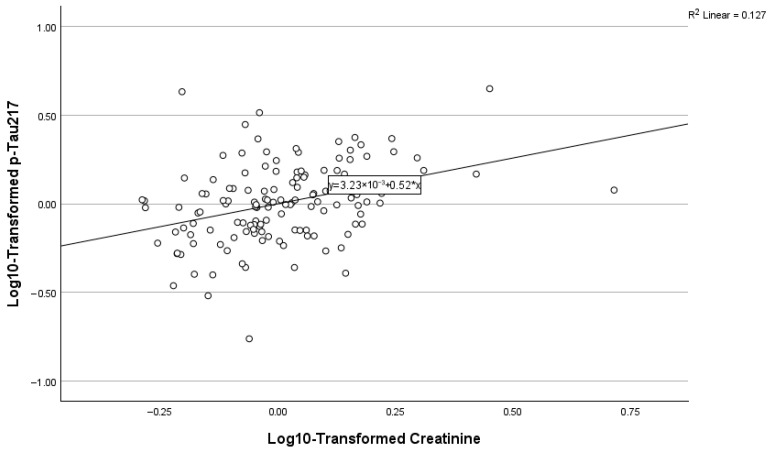
Relationship between p-Tau217 and creatinine.

**Figure 3:**
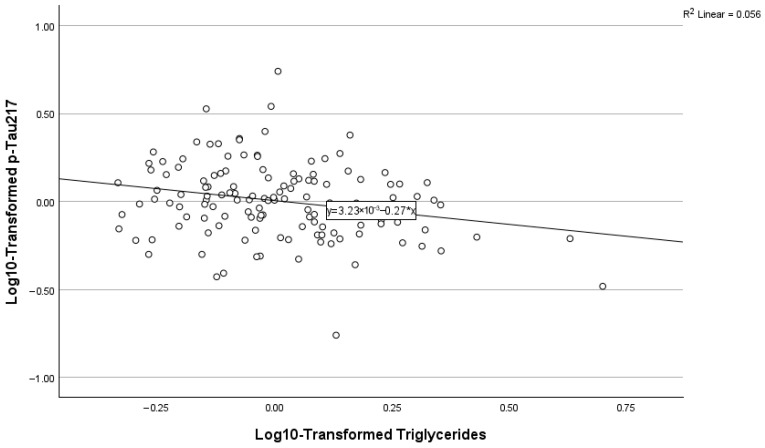
Relationship between p-Tau217 and triglycerides.

As depicted in Table 3 and Figure 1, Figure 2 and Figure 3 below, amyloid PET positivity was the strongest predictor of p-Tau-217, accounting for approximately 28.8% of the variance (ΔR^2^ = 0.288, β = 0.409, *p* < 0.001). The addition of creatinine significantly improved model fit. High creatinine was associated with high p-Tau217 concentrations (cumulative R^2^ = 0.392, β = 0.331, *p* < 0.001). In addition, levels of triglycerides were inversely related to p-Tau217 concentrations (β = −0.185, *p* = 0.007). This final model explained approximately 43% of the variance in plasma p-Tau217 (cumulative R^2^ = 0.435).

### 3.7. Predictors of Plasma GFAP

**Figure 4:**
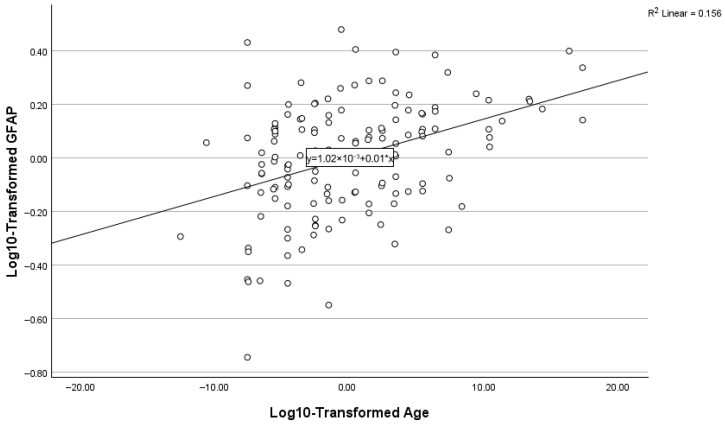
Relationship between GFAP and age.

**Figure 5:**
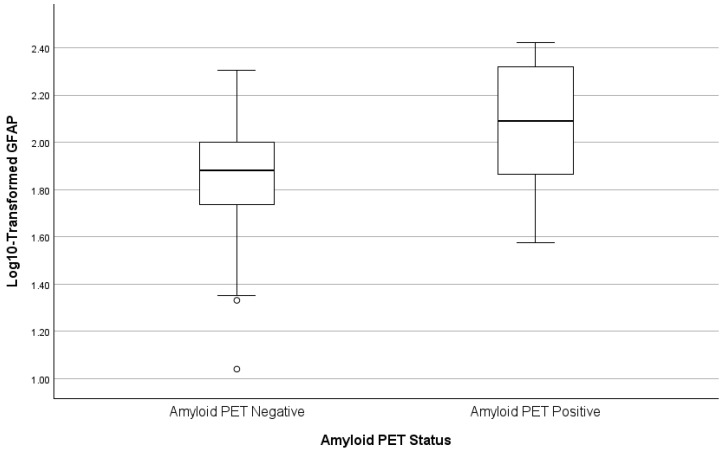
Box plots of GFAP by amyloid PET status.

**Figure 6:**
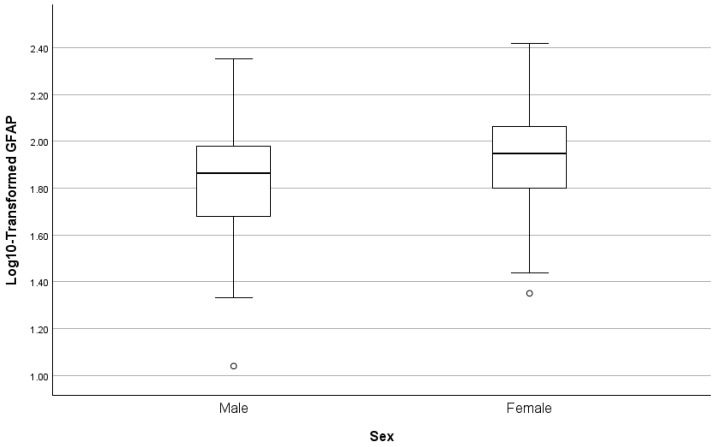
Box plots of GFAP by sex.

Results of the stepwise regression predicting log10-transformed GFAP are presented in Table 4 and Figure 4, Figure 5 and Figure 6. Age was the dominant initial predictor (β = 0.404, *p* < 0.001), explaining more than 16% of the total variance. Amyloid PET positivity was also significantly associated with elevated GFAP (cumulative R^2^ = 0.261, β = 0.320, *p* < 0.001). In the final step, sex contributed independent explanatory power (β = 0.242, *p* = 0.001). This final model explained approximately 32% of the variance in plasma GFAP (cumulative R^2^ = 0.320).

### 3.8. Predictors of Plasma NfL

**Figure 7:**
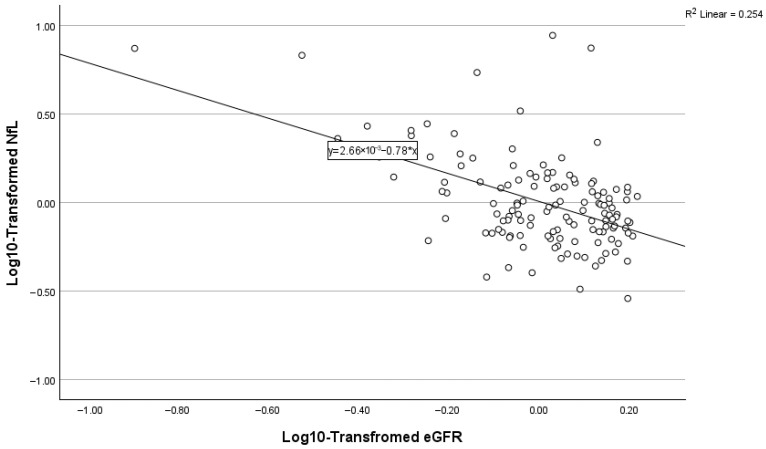
Relationship between NfL and eGFR.

**Figure 8:**
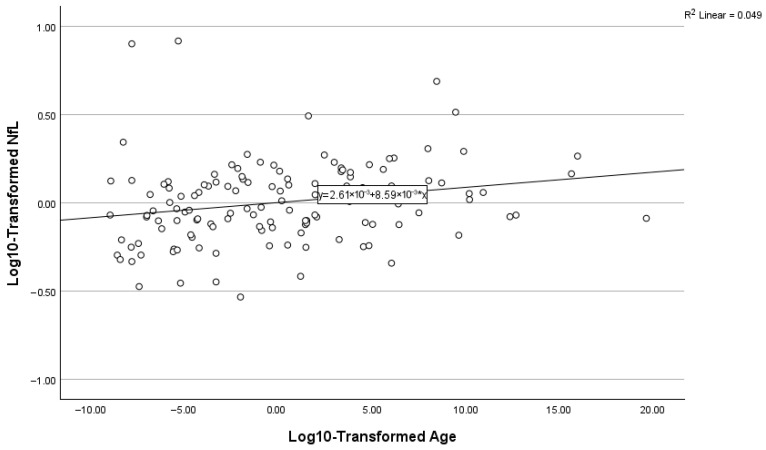
Relationship between NfL and age.

**Figure 9:**
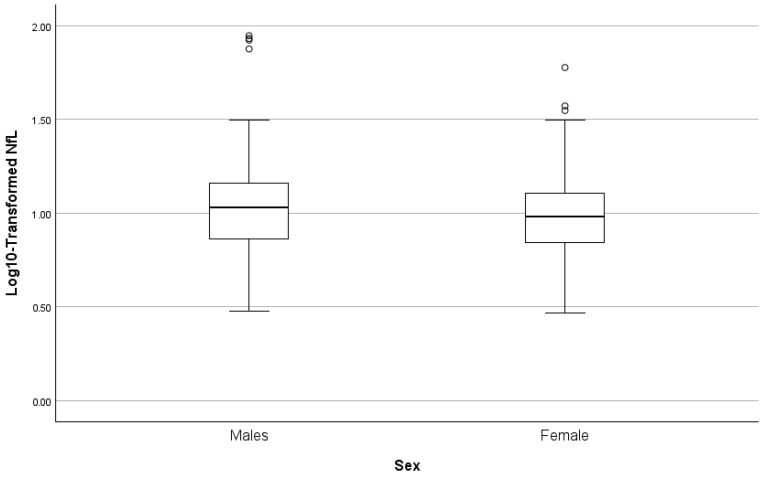
Box plots of NfL by sex.

Stepwise regression models predicting log10-transformed NfL are summarized in Table 5 and Figure 7, Figure 8 and Figure 9. A lower level of renal function (eGFR) was the strongest predictor (β = −0.519, *p* < 0.001), explaining approximately 27% of the variance in NfL. Age was next (cumulative R^2^ ≈ 0.306, β = 0.180, *p* = 0.018). In Step 3, the addition of sex resulted in a modest but significant increase in explained variance (ΔR^2^ = 0.022). Female sex (β = −0.155, *p* = 0.035; reference = male) was independently associated with log_10_-transformed NfL, with females exhibiting lower NfL levels relative to males. This final model explained approximately 33% of the variance in plasma NfL (cumulative R^2^ = 0.325).

## 4. Discussion

Plasma biomarkers are increasingly used to detect and monitor AD and ADRD in older adults. However, their interpretation may be complicated by biological and systemic factors that do not necessarily reflect central nervous system pathology. Our investigation of a large number of community-dwelling Black/African American older adults yielded several key findings regarding potential physiological influences on plasma-based biomarkers. First, consistent with the prior literature, the concentration of plasma p-Tau217 was most strongly correlated with amyloid PET positivity. This reinforces the diagnostic potential of p-Tau217 as a surrogate for cerebral amyloidosis in this population. Furthermore, the significant intercorrelations observed between p-Tau217, GFAP, and NfL indicate shared variance that likely reflects the effects of neurodegenerative processes.

In this study, we studied a large number of community-dwelling B/AA older adults who are at increased risk for cognitive impairment, dementia, and other medical conditions that may influence AD and neurodegenerative blood results. A critical observation in this study was the substantial role of renal function in the prediction of plasma biomarker levels, independent of amyloid pathology. This is supported by the emerging literature, which suggests that measuring the impact of kidney function may be particularly prudent for Black and African American older adults who experience a disproportionately higher prevalence of chronic kidney disease and other medical comorbidities [29]. While amyloid PET status was the primary driver of p-Tau217 (explaining 31.4% of variance), creatinine was also a significant co-predictor. Further, rather than amyloid PET status, eGFR emerged as the strongest predictor of NfL levels (26.9% of variance), outweighing the influence of age or sex. These results suggest that in individuals with reduced renal clearance, a condition prevalent in B/AA communities, plasma concentrations of p-Tau217 and NfL may be artificially elevated, potentially leading to false-positive interpretations of neurodegenerative risk if not adjusted for kidney function.

In contrast to p-Tau217 and NfL, GFAP levels were predominantly driven by age (16.3% of variance) and female sex, and to a lesser degree, amyloid PET positivity. This indicates that GFAP may be more sensitive in B/AA adults to age-related and potentially sex-related differences in astrogliosis or inflammation rather than issues associated with measures of renal function/clearance.

Finally, we noted a modest but significant inverse relationship between triglycerides and p-Tau217 (3.4% of variance), which was intriguing yet consistent with the emerging literature that suggests that lower triglyceride levels may be associated with increased dementia risk in population-based studies [30,31]. Proposed mechanisms include that low triglycerides may reflect frailty and poor nutritional status, both of which are independently associated with dementia risk. In addition, triglyceride fractions may reflect lipid components, including polyunsaturated fatty acids, that have been implicated in AD-related pathways [32]. The observed associations for triglycerides (ΔR^2^ = 3.4%), while statistically detectable within this sample, have small effect sizes and may not translate into clinically meaningful differences at the individual level. Accordingly, replication in independent and larger cohorts is necessary to determine the robustness, stability, and generalizability of these findings. While the biological mechanism requires further exploration, this finding raises the possibility that lipid metabolism may possibly modulate the stability of phosphorylated tau in the periphery, adding another layer of complexity to biomarker interpretation in patients with metabolic comorbidities. Given the small proportion of explained variance, this association should be interpreted cautiously and warrants replication.

In summary, while plasma p-Tau217 served as a robust indicator of amyloid pathology in B/AA older adults, its concentration, alongside NfL’s, was significantly associated with measures of renal function and clearance. Conversely, GFAP levels were primarily driven by age and sex. These results underscore that systemic health and demographic factors contribute meaningfully to plasma biomarker variability and should be considered when interpreting results.

Given the current lack of validated biomarker cut-points for community-dwelling B/AA populations, these findings underscore the importance of accounting for age, sex, and renal function when interpreting markers of phosphorylated tau, astroglial activation (GFAP), and axonal injury (NfL). Current commercially available and direct-to-consumer biomarker platforms do not consistently incorporate these factors, which may limit diagnostic sensitivity and specificity in populations with a high burden of comorbidities.

A primary strength of this investigation is the deep phenotyping of a large, community-dwelling cohort of B/AA older adults. Unlike many AD studies that rely on clinical convenience samples, our participants were recruited from the community rather than specialty memory centers, enhancing the generalizability of our findings to the broader B/AA population. Furthermore, the inclusion of multimodal biomarkers including amyloid PET, plasma-based AD and neurodegenerative markers (p-Tau217, GFAP, NfL), APOE ε4 genotyping, and comprehensive clinical metabolic, lipid and renal profiles allowed for a more objective examination of how systemic health factors interact with the biomarkers under study.

Despite these strengths, several limitations warrant consideration. First, while we accounted for systemic comorbidities, cerebrovascular co-pathologies (e.g., small vessel disease, strategic infarctions) which might be predictive of plasma biomarkers of AD and neurodegeneration in the blood were not assessed, nor accounted for. As it was beyond the scope of the current investigation, we did not examine vascular pathology such as strategic infarcts or small vessel disease that may be related to AD pathology. Moving forward, our research group is expanding the imaging methodology in this unique cohort to incorporate neuroimaging metrics of small vessel disease and other cerebrovascular pathologies which will enable independent evaluation of the association of cerebrovascular pathology to plasma biomarkers and to amyloid PET. Second, while certain markers were analyzed alongside PET, which remains the gold standard for defining pathological AD, future biomarker studies should be studied and validated against longitudinal PET and tau imaging. Amyloid PET positivity in this study was determined using expert visual reads rather than SUVR-based cut-points. This approach was necessary because over 20% of participants were ineligible for MRI, which is required for SUVR quantification from T1-weighted imaging. Expert visual interpretation is widely used in both clinical and research settings and is considered a reliable method for classifying amyloid status [28]. However, we acknowledge that the absence of SUVR-based thresholds, and the limited validation of such cut-points in Black/African American populations, may introduce some variability in classification and could potentially influence the observed associations. Third, the relatively small number of amyloid PET positive participants (n = 26) precluded meaningful subgroup analyses (e.g., stratification by CKD stage or diabetes severity), thereby limiting our ability to evaluate potential effect modification within biologically and clinically relevant subgroups. Also, while our cohort is community-based and recruited in South Florida, direct comparison of comorbidity prevalence (e.g., CKD, diabetes) with national estimates in older Black/African American populations is limited by substantial heterogeneity across studies in sampling frames, recruitment methods, and ascertainment of medical conditions.

As a result, we cannot determine with certainty whether the comorbidity profile observed in our sample differs from national patterns. The South Florida catchment area is characterized by marked socioeconomic heterogeneity, diverse immigration histories, and variable access to healthcare, all of which may shape underlying disease prevalence and observed biomarker relationships. These regional and sociodemographic factors may therefore limit generalizability to other geographic settings. Thus, multi-site studies that systematically evaluate regional and structural determinants of comorbidity burden and their potential impact on AD/ADRD biomarker associations will be essential for establishing findings that are robust, generalizable, and clinically actionable across diverse populations.

## 5. Conclusions

Collectively, the present findings suggest that while plasma biomarkers offer a cost-effective, non-invasive alternative to traditional diagnostics, their concentrations in B/AA older adults reflect more than isolated brain pathology associated with AD and ADRD. Specifically, levels of p-Tau217 and NfL appear to be influenced by systemic health factors, including renal function, age, and sex. To support diagnostic accuracy and reduce the potential for misinterpretation of elevated biomarker levels, clinical interpretation would benefit from approaches that extend beyond fixed, raw thresholds and instead incorporate more nuanced, health-informed models. Such a multimodal framework may be particularly important for community-dwelling B/AA populations, who experience a disproportionate burden of both dementia and systemic comorbidities, yet remain underrepresented in biomarker research and less likely to seek care in specialty memory clinics.

Finally, as these systemic factors likely influence the longitudinal trajectories of AD and neurodegenerative markers, our ongoing prospective studies will investigate how renal, demographic, and other variables relate to biomarker changes over time in populations with a high comorbidity burden. Thus, accounting for these covariates is an essential step toward improving the diagnostic specificity, and interpretability, of blood-based biomarkers in both clinical and research settings.

## Figures and Tables

**Table 1:** Clinical characteristics, plasma biomarkers and renal and metabolic values of the study population (N = 141).

Variable	Mean (SD)	Range
Demographic and Cognition		
Age, years	68.2 (6.0)	59.0–88.0
MMSE Score	27.3 (2.4)	16.0–30.0
Plasma Biomarkers		
p-Tau217, pg/mL	0.37 (0.30)	0.07–2.07
GFAP, pg/mL	91.8 (51.0)	11.0–263.1
NfL, pg/mL	13.9 (14.2)	2.9–88.4
Lipid, Metabolic, and Renal Values		
Triglycerides, mg/dL	104.8 (60.8)	41.1–490.7
Total Cholesterol, mg/dL	173.4 (42.0)	85.5–326.1
LDL Cholesterol, mg/dL	93.71 (35.3)	17.0–206.0
Hemoglobin Alc	6.01 (1.02)	2.0–10.0
BUN, mg/dL	16.4 (8.4)	2.0–79.0
Creatinine, mg/dL	1.08 (0.59)	1.0–6.0
eGFR, mL/min/1.73 m	78.7 (24.1)	9.5–121.4

Abbreviations: BUN, blood urea nitrogen; eGFR, estimated glomerular filtration rate; GFAP, glial fibrillary acidic protein; MMSE, Mini-Mental State Examination; NfL, neurofilament light chain; p-Tau217, phosphorylated tau 217.

**Table 2:** Distribution of categorical demographic and clinical variables.

Characteristic	Frequency (n = 141)	Percentage (%)
Sex		
Female	82	58.6
Male	58	41.4
ApoE4 Status		
Carrier (Positive)	50	35.7
Non-Carrier (Negative)	90	64.3
Amyloid PET Status		
Positive	26	18.4
Negative	115	81.6

Note: Sex data were missing for 1 participant; percentage is calculated based on valid cases (n = 140).

**Table 3:** Stepwise linear regression analysis for predictors of p-Tau217 (N = 131).

Model and Variable	Standardized Coefficient (β)	95% CI	*p* Value	ΔR^2^	Cumulative R^2^
Step 1				0.314	0.314
Amyloid PET Positive	0.560	0.303 to 0.514	<0.001		
Step 2				0.088	0.401
Amyloid PET Positive	0.489	0.255 to 0.458	<0.001		
Creatinine	0.305	0.293 to 0.785	<0.001		
Step 3				0.034	0.435
Amyloid PET Positive	0.475	0.248 to 0.446	<0.001		
Creatinine	0.293	0.277 to 0.757	<0.001		
Triglycerides	−0.185	−0.460 to −0.076	0.007		

**Table 4:** Stepwise linear regression analysis for predictors of GFAP (N = 131).

Model and Variable	Standardized Coefficient (β)	95% CI	*p* Value	ΔR^2^	Cumulative R^2^
Step 1				0.163	0.163
Age	0.404	0.010 to 0.022	<0.001		
Step 2				0.098	0.261
Age	0.341	0.007 to 0.020	<0.001		
Amyloid PET Positive	0.320	0.102 to 0.290	<0.001		
Step 3				0.058	0.320
Age	0.342	0.008 to 0.019	<0.001		
Amyloid PET Positive	0.339	0.117 to 0.298	<0.001		
Sex	0.242	0.047 to 0.186	0.001		

**Table 5:** Stepwise linear regression analysis for predictors of NfL (N = 131).

Model and Variable	Standardized Coefficient (β)	95% CI	*p* Value	ΔR^2^	Cumulative R^2^
Step 1				0.269	0.269
eGFR	−0.519	−1.067 to −0.593	<0.001		
Step 2				0.031	0.301
eGFR	−0.486	−1.014 to −0.540	<0.001		
Age	0.180	0.001 to 0.015	0.018		
Step 3				0.024	0.325
eGFR	−0.495	−1.026 to −0.558	<0.001		
Age	0.175	0.001 to 0.015	0.020		
Sex	−1.55	−0.166 to −0.006	0.035		

## Data Availability

The datasets presented in this article are not readily available because the data are part of an ongoing study. Requests to access the datasets should be directed to Dr. Rosie Curiel Cid at rcuriel2@med.miami.edu.
